# Enzyme renaturation to higher activity driven by the sol-gel transition: Carbonic anhydrase

**DOI:** 10.1038/srep14411

**Published:** 2015-09-23

**Authors:** Vladimir V. Vinogradov, David Avnir

**Affiliations:** 1 Laboratory of Solution Chemistry of Advanced Materials and Technologies, ITMO University, St. Petersburg, 197101, Russian Federation; 2 Institute of Chemistry and the Center for Nanoscience and Nanotechnology, the Hebrew University of Jerusalem, Jerusalem 91904, Israel

## Abstract

We describe a so-far unknown route for renaturing denatured enzymes, namely subjecting the denatured enzyme to an oxide sol-gel transition. The phenomenon was revealed in a detailed study of denatured carbonic anhydrase which was subjected to an alumina sol-gel transition, up to the thermally stabilizing entrapment in the final xerogel. Remarkably, not only that the killed enzyme regained its activity during the sol-gel process, but its activity increased to 180% of the native enzyme. To the best of our knowledge, this is the first report of *enhanced* activity following by renaturing (a “Phoenix effect”). Kinetic study which revealed a five-orders of magnitude (!) increase in the Arrhenius prefactor upon entrapment compared to solution. Circular dichroism analysis, differential scanning calorimetry, zeta potential analyses as well as synchronous fluorescence measurements, all of which were used to characterize the phenomenon, are consistent with a proposed mechanism which is based on the specific orienting interactions of the active site of the enzyme with respect to the alumina interface and its pores network.

Enzymes entrapments in ceramic sol-gel matrices proved in the past 25 years to be one of the more efficient methods for their heterogenization^1^,^2^, for their protection from heat^3^ and for their protection from harsh chemicals^4^; the literature on this methodology is voluminous^5^,^6^,^7^. We now report on two new related features of this biomaterials approach, observed for carbonic anhydrase and sol – gel derived alumina, which to the best of our knowledge has not been described before neither in the context of biomaterials not in the context of enzymes renaturation: First, the ability of the sol-gel process to renature a denatured enzyme upon entrapment of the denatured form; and second, achieving significantly higher activity of the renatured enzyme compared to the activity of the native enzyme in solution. Proteins are easily denatured by shifting away from their optimal environmental conditions; the refolding of the polypeptide chain back to its natural state is by far more difficult and demanding. However, the importance of affecting and controlling that process cannot be exaggerated, as it bears on issues such as the toxicity and pathogenic action of denatured proteins and prion diseases (Alzheimer, Huntington and Parkinson diseases^8^). Two of the main obstacles for denatured proteins to refold back correctly are the persistent exposure to denaturing chemicals, and the tendency of the (denatured) macromolecules to aggregate. Aggregation greatly hinders the motional freedom of the polypeptide chain in its screening of the various folded options, including the natural one. One of the main *in-vitro* tools for renaturation has been the use of surfactants and other small molecules^9^,^10^,^11^. Another approach has been the use of adsorbents such as zeolites^12^ and silica^13^, for separating the protein molecules from the denaturing chemicals^14^. Of relevance to this report are the unfolding/refolding studies of proteins entrapped in their native (non-denatured) form within sol-gel matrices^15^; a main motivation in these studies has been to take advantage of the slow-down of the unfolding-refolding kinetics under these conditions, which provided valuable insight into these transitions.

Previous studies by us^16^,^17^,^18^,^19^ and by other laboratories^3^, which indicated exceptional thermal stability of sol-gel entrapped proteins – particularly alumina entrapped proteins^16^ – directed our attention to the possibility that the sol-gel process itself can be used for renaturation of thermally deactivated proteins; we hypothesized that the aggregation of the sol particles to the porous gel, may have traits which can assist refolding – proof of this hypothesis is represented below and described for the first time. It should be noted that renaturation of thermally de-activated enzymes^8^, has been much less explored than the denaturation of chemically denatured proteins; this is so because the majority of chemically denaturing studies involved reversible interruption with hydrogen bonds, while thermal denaturation has the potential for being irreversible.

The enzyme which we explored in detail for this study is bovine carbonic anhydrase (CAB) – a most-common enzyme for renaturation studies. The sol-gel process which proved to be suitable for our purpose is the bio-friendly ultrasound-assisted synthesis we developed in a series of recent papers^16^,^17^,^18^. By this procedure, the obtained sol is of highly pure Boehmite nanoparticles, dispersed in pure water. The first stage had been the evaluation of the thermal stability of alumina entrapped native CAB – it was found to be exceptional; here are the details: Fig. 1a follows the ellipticity changes (of the 215 nm helical peak) as a function of temperature: It is seen that while in solution the ellipticity drops sharply at ~68 °C, the signal of CAB from the alumina entrapped biocomposite, CAB@alumina, is unaffected at that temperature and remains stable at least until 90 °C. Clearly, while the free enzyme undergoes conformational changes above 68 °C in solution, this is avoided in the entrapped form at that temperature and even at higher temperatures, as seen in the figure. Yet another indication for the enhanced stability comes from differential scanning calorimetry (DSC) analysis: It is seen (Fig. 1b) that the denaturation temperature of CAB is shifted to a higher temperature by 51 °C (!) when entrapped within the alumina matrix, that is, from ~68 °C to a staggering ~119 °C. These observations are in a good agreement with the observed comparison of activities of free and entrapped CAB, first at 37 °C and then after heating to 70 °C for 10 min (Fig. 1c): While the activity of the free CAB dropped to very small residual level, the entrapped enzyme retained 88% of its initial activity.

Having the results of Fig. 1 at hands, we move to our main goal, namely the denaturing-renaturation experiments: Thermal denaturation of CAB was first carried out at 65 °C (based on Fig. 1a,b) for 10 min in a 16 mM Tris-sulfate buffer (pH 7.5). The renaturation was affected by subjecting a buffered solution of the denatured CAB to a freshly prepared alumina sol which was left to gel spontaneously at room temperature for 24 h (see Experimental Details). Comparative CD spectra of the solutions of the native CAB and of the thermally denatured one (black and blue, respectively, in Fig. 2) clearly show the change in the protein structure: The negative peak at 215 nm which is characteristic of the β-sheet structure present in the enzyme is replaced by a large negative peak at 207 nm and a shoulder at 220 nm, consistent with the formation of non-native α-helices^16^. Upon subjecting the denatured CAB to the alumina sol-gel process, CD (red line, Fig. 2) shows reconstruction of the original structure, down to fine finger-print details of the spectra.

The denaturation detected in the CD spectrum, is indeed reflected in the activity of the enzyme, as shown in Fig. 3 (red and green columns). It is seen in the blue column of that figure that subjecting the denatured CAB to the entrapping sol-gel process, resulted in activity which is not only restored, but *surpasses the native activity to a level of 180*% (average of three experiments: 167, 164 and 209%).

We propose that the CAB renaturation follows a process described in Fig. 4: The exposed residues of the unfolded protein (formed after thermal denaturation, stage I, Fig. 4) bind to the alumina nanoparticles (stage II) mainly through ionic and polar hydrogen bonds between the surface moieties of the alumina and the polar and ionic amino acid moieties of the protein (see potential values below), thus diminishing hydrophobic aggregation interactions^20^. This is then followed by the polymerization of the sol particles and the formation of a three-dimensional gel framework (stage III), that on one hand suppresses protein aggregation, but on the other hand, offers enough aqueous space for motions of the polypeptide chain in its search for the optimal folding. The final stage IV is the entrapment of the refolded protein in the solid bio-xerogel cages.

Dynamic light scattering proof of the de-aggregation of thermally denatured CAB is provided in Fig. 5: Whereas heating CAB to 65 °C leads to strong aggregation, increasing the average size of CAB aggregates from 105 nm to 100 μm, adding alumina sol to the denatured CAB reverses this process, forming two types of orders of magnitude smaller aggregates at 150 nm and 1056 nm (pure alumina sol nanoparticles have an average hydrodynamic size of 80 nm). The driving force for the interaction between the alumina interface and the protein which leads to the de-aggregation is the electrostatic force between positively charged alumina (+45 mV) and the negatively charged CAB (−12 mV). Indeed, following this interaction (after mixing) the potential of the bio-colloid changes to +32 mV.

In the proposed mechanism the early interactions between the denatured CAB and the alumina sol are not the crucial step, and this is proven by synchronous fluorescence measurements (Fig. 6): The shifts in the positions of the maxima (blue shifts) clearly seen for CAB after denaturation are practically the same before and after the addition of the alumina sol. Absence of renaturation by the sol itself was also confirmed by reactivity analysis: only 15% (±3%) of activity was observed after mixing alumina nanoparticles with the denatured CAB. Thus, the crucial step for the renaturation is the build-up of the porosity (details on porous structure are presented in experimental details the gel itself as a supportive scaffold, as was clearly demonstrated in the CD spectra (Fig. 2) and in the kinetics analysis (Fig. 3).

We attribute the enhancement of CAB activity to the combined effects of the directional exposure of the active site of the enzyme into the pore space, and of the efficient directing of the substrate molecules to the active site of the enzyme. The first of these effects is the preferred orientation of the renatured CAB active site away from the alumina interface and into the pore volume, and we propose that it is due to the following mechanism: We recall that CAB operates through a zinc-histidine complex located in its active site^21^. Entrapment of the native form of the enzyme leads to a distribution of orientations of the CAB molecules, some of which have their active site open to the pore network and therefore accessible to reaction, while others are not – this is well documented in the literature of sol-gel entrapped enzymes^22^. But when *unfolded denatured* CAB is entrapped, an interesting situation emerges: The Zn cation is complexed to three histidine residues (H94, H96 and H119^21^), all of which comprise a heavily positive charged site: the metal cation and the protonated non-complexing nitrogen atoms of the imidazole-ring moieties of histidine (the pKa is very high – 14.5 and 7.05 for the conjugated acid^23^). The protein is thus oriented during the refolding process so that the positively charged alumina wall (zeta potential +45 mV) pushes away the active site into the pore volume, while interacting with the protein through the negatively charged aspartic and glutamic acids residues which are not part of the active site but are abundant in CAB molecule^24^.

The second effect, namely the pronounced direction of the substrate molecules to the active site was proven by a detailed Arrehnius analysis, which compared the free, native-entrapped, and denatured-entrapped CAB molecules (Fig. 7). It is first seen that the Arrehnius plots have two zones: The negative slopes (the right side) which reflects the increase in activity with temperature, and the apparent negative activation energies on the left, which reflect the thermal destruction of the enzyme – we concentrate of the right side: We first see that the activation energies for the native-entrapped and the renatured entrapped CAB are quite similar, and this is yet another indication of the successful renaturing process. Note also that the activation energy for the free CAB is much lower - the low activation energy is a reflection of the known high efficiency of this enzyme^25^, and the higher activation energies for the entrapped enzymes reflect the diffusional barriers of the entering substrate molecules (sol-gel entrapped enzymes are known for that property^26^). But the real eye opener comes from the comparative analysis of the Arrehnius pre-factors: While for the free enzyme it is 0.06 1/sec, for the entrapped renatured CAB it *is more than five orders of magnitudes higher – 8654 1/sec!*; that is, the rate of successful collisions between the enzyme and the substrate, once it reaches the enzyme, is by far more efficient for the entrapped enzyme – it is a pore-directing effect, which replaces random collisions with the enzyme all around it, by unidirectional collisions (like a pointed machine-gun) dictated by the pore channel leading to the active site. Furthermore, it is expected that if the first effect described above indeed operates, then the prefactor for the entrapped native enzyme will be lower, because its entrapment is not accompanied by re-orientation; this is indeed the case – the prefactor in this case is lower: 7758 1/sec.

To conclude, all of the analyses detailed above are best unified into a consistent picture by which the observation that CAB is not only efficiently renatured but also reaches higher activity compared with the native enzyme in solution, is best interpreted by a renaturation mechanism that is assisted by the gradual forming of alumina cages within which the enzyme is finally entrapped, in an orientation which exposes its active site to the pore network, which in turn, directs the substrate to that site. As we could not find any previous report on renaturation which leads to enhancement of activity, we follow the cross-culture mythology which uses the Phoenix legend as a symbol for rebirth sometimes into even stronger self, and term this phenomenon, the *Phoenix effect*. We believe that the observation of this effect on CAB indicates a realistic possibility of generalization of such matrix-assisted renaturations to other proteins.

## Experimental Details

### Chemicals

Aluminum isopropoxide, carbonic anhydrase from bovine erythrocytes (CAB, cat. No. C393), p-nitrophenyl acetate (pNPA) were all obtained from Sigma-Aldrich. Tris-sulfate buffer was prepared from respective solution with the desired volumes of 1.0 M NaOH.

### Thermal denaturation CAB

Thermal denaturation of CAB was conducted by denaturing 2 mL of CAB (80 U/mL) at 65 and 70 °C for 10 min in 16 mM Tris-sulfate buffer, pH 7.5. Heating the CAB solution was carried out at a continuous rate of 0.5 °C/min.

### Renaturation and entrapping the CAB within alumina

Boehmite alumina sol was prepared in a bio-friendly ultrasonic procedure as previously described^10^. Such sols provide alumina xerogel which has specific surface area 405 m^2^/gr, average pore size 3.0 nm and pore volume 0.334 cm^3^/gr, as determined from nitrogen adsorption. For renaturation of the denatured enzyme or for the entrapment of the native enzyme, a mixture of 100 μL of 16 mM Tris-sulfate buffer, pH 7.5, and 400 μL of freshly prepared alumina sol was transferred to a cuvette and then 200 μL of free or denatured CAB (80 U/mL) was added. Ten minutes later the sol was left in vacuum desiccator at room temperature for 24 h.

### Enzymatic activity of free and entrapped CAB

Prior to analysis the bioactive dried hybrids were left for incubation with 0.5 ml of 16 mM Tris-sulfate buffer (pH 7.5) at 37 °C for 30 min. Then they were covered with 2.0 mL of p-nitrophenyl acetate solution (prepared by mixing 0.6 mg/mL of pNPA with 16 mM Tris-sulfate buffer, pH 7.5). The enzymatic activity was measured by following at 400nm the yellow p-nitrophenol formed by the hydrolysis of pNPA. For comparative analysis, free CAB, 20 μL (80 U/ml) in 2.0 mL of 16 mM Tris-sulfate buffer, pH 7.5 was reacted similarly to the entrapped enzyme (and to compensate for the slower reactivity of the entrapped enzymes, ×10 lower concentrations of the free enzymes were taken). The activity of entrapped native CAB was compared with activity of the entrapped renatured CAB. For reproducibility measurements, at least three measurements under the same conditions were carried out.

### Characterization techniques

The spectral analysis of enzymatic activity was carried out using HP 8453 Diode Array spectrophotometer. CD spectra were recorded on a J-810 instrument. The measurements of solutions were taken using a 2-mm path length cuvette and coated slides were used for the entrapped proteins at 20 °C. Thermal stability studies were performed between 20 and 90 °C, at a constant heating rate of 3 °C/min. Dynamic light scattering measurements were carried out using Compact Z Photocor Instrument. Synchronous fluorescence spectra were recorded with Cary Eclipse fluorimeter.

## Additional Information

**How to cite this article**: Vinogradov, V. V. and Avnir, D. Enzyme renaturation to higher activity driven by the sol-gel transition: Carbonic anhydrase. *Sci. Rep.* **5**, 14411; doi: 10.1038/srep14411 (2015).

## Figures and Tables

**Figure 1:**
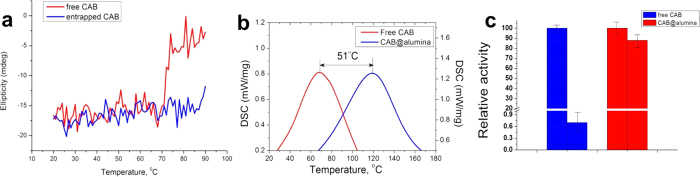
(**a**) Ellipticity of CAB as a function of the heating temperature (heating rate of 1 °C min^−1^), monitored at the 215 nm helical peak: It is seen that while in solution the ellipticity drops sharply at ~68 °C, the signal of CAB@alumina is unaffected at that temperature, and remains stable at least until 90 °C. (**b**) DSC analysis of CAB@alumina: An up-shift of 51 °C in the denaturation temperature is observed for CAB@alumina (right curve), compared with free CAB (left curve). (**c**) Comparison of the enzymatic activity of free (blue columns) and entrapped (red columns) CAB at 37 °C (left blue and red) and after heating to 70 °C (right blue and red; the activities are set to relative 100% - see Experimental Details).

**Figure 2:**
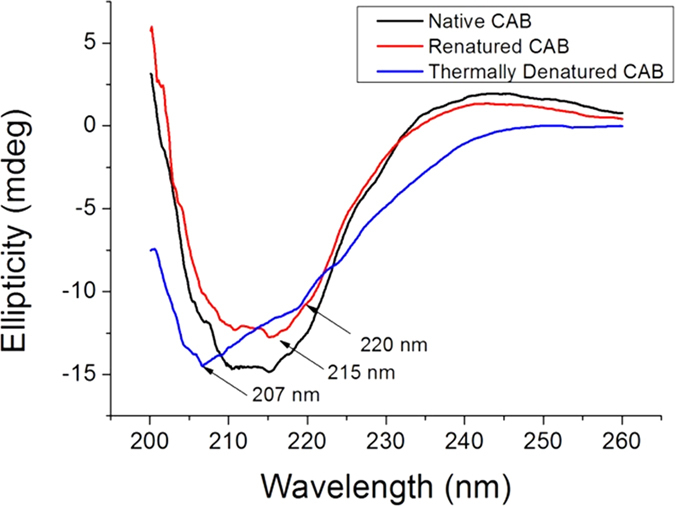
Solution CD spectra of the native and of the thermally denatured CAB (black and blue), and the CD spectrum of the refolded CAB entrapped in alumina (red).

**Figure 3:**
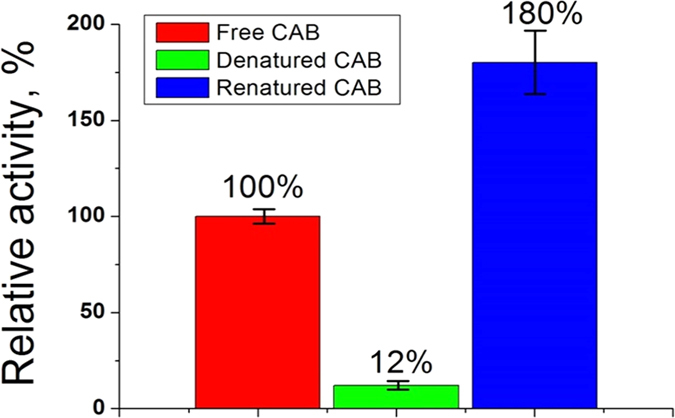
The original activity of CAB in solution (red) greatly diminished upon denaturation in solution at 65 °C (green). The denatured enzyme is renatured to much higher activity (blue) upon entrapment in the alumina sol-gel matrix.

**Figure 4:**
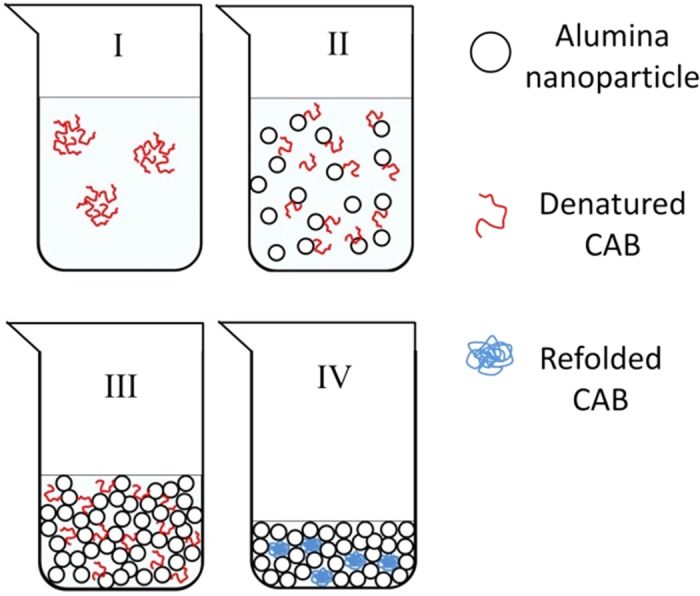
Suggested scheme of the sol – gel transition assisted CAB refolding. (Made by V. Vinogradov).

**Figure 5:**
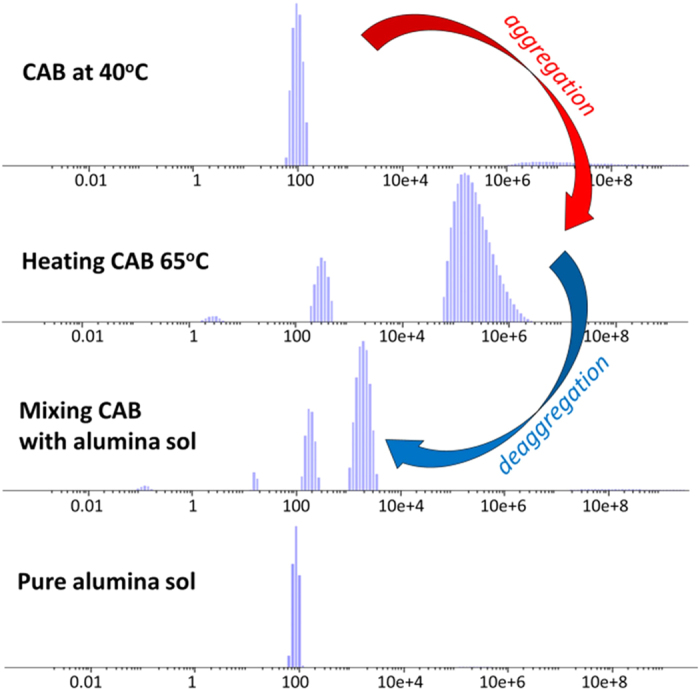
The hydrodynamic particle sizes of free CAB in Tris-sulfate buffer at 40 °C, of free CAB after heating to 65 °C, of denatured CAB molecules after binding with alumina nanoparticles, and of alumina sol.

**Figure 6:**
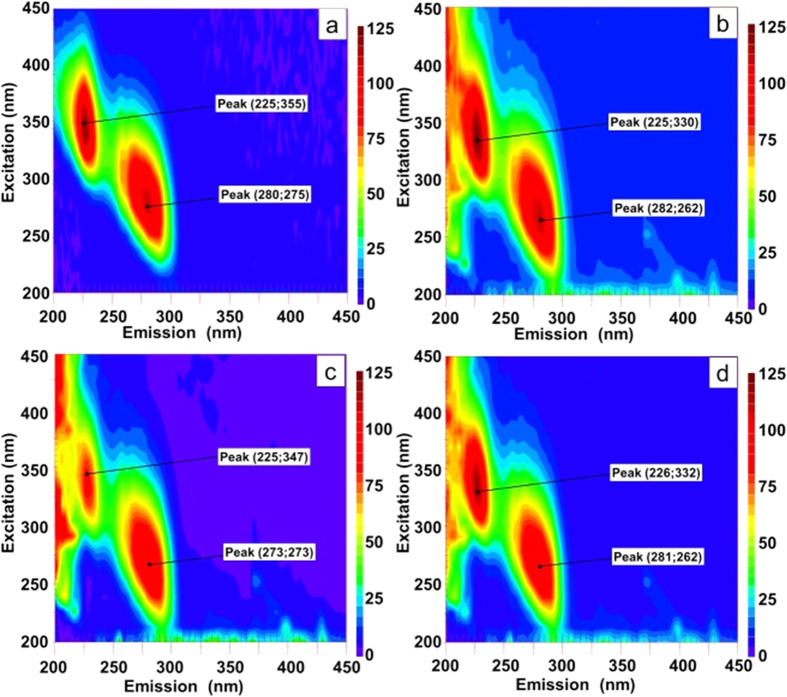
Synchronous fluorescence spectra of (a) native CAB, (b) CAB denatured by heating to 65 °C, (c) native CAB mixed with alumina, and (d) denatured CAB mixed with alumina.

**Figure 7:**
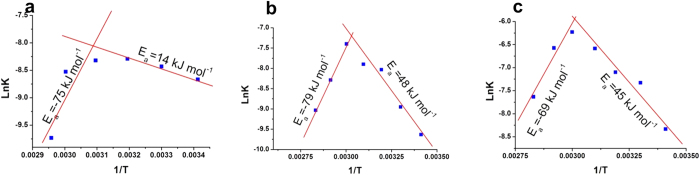
Arrhenius plot for pNPA hydrolysis catalyzed by free native (a), native entrapped (b), refolded entrapped (c) CAB. There are temperature switch points, above which the apparent activation energy becomes negative. The calculated Arrhenius pre-factors are 0.06, 8654, 7758 sec^−1^ respectively. Temperatures in Kelvin.
